# Supplementary material for: Signatures of VH1-69-derived hepatitis C virus neutralizing antibody precursors defined by binding to envelope glycoproteins
Source: Nat Commun. 2023 Jul 7;14:4036. doi: 10.1038/s41467-023-39690-0 (PMC10328973; doi:10.1038/s41467-023-39690-0)

Signatures of V_H*1-69*-derived hepatitis C virus neutralizing antibody precursors defined by binding to envelope glycoproteins

Joan Capella-Pujol[1,2], Marlon de Gast[1,2], Laura Radić[1,2], Ian Zon[1,2], Ana Chumbe[1,2], Sylvie Koekkoek[1,2], Wouter Olijhoek[1,2], Janke Schinkel[1,2], Marit J. van Gils[1,2], Rogier W. Sanders[1,2,3*], Kwinten Sliepen[1,2*]

[1]Department of Medical Microbiology and Infection Prevention, Laboratory of Experimental Virology, Amsterdam UMC, University of Amsterdam, 1105 AZ Amsterdam, Netherlands.

[2]Amsterdam Institute for Infection and Immunity, Infectious Diseases, 1105 AZ Amsterdam, Netherlands.

[3]Department of Microbiology and Immunology, Weill Medical College of Cornell University, New York, NY 10065, USA.

* Corresponding authors: k.h.sliepen@amsterdamumc.nl, r.w.sanders@amsterdamumc.nl

Supplementary Figures

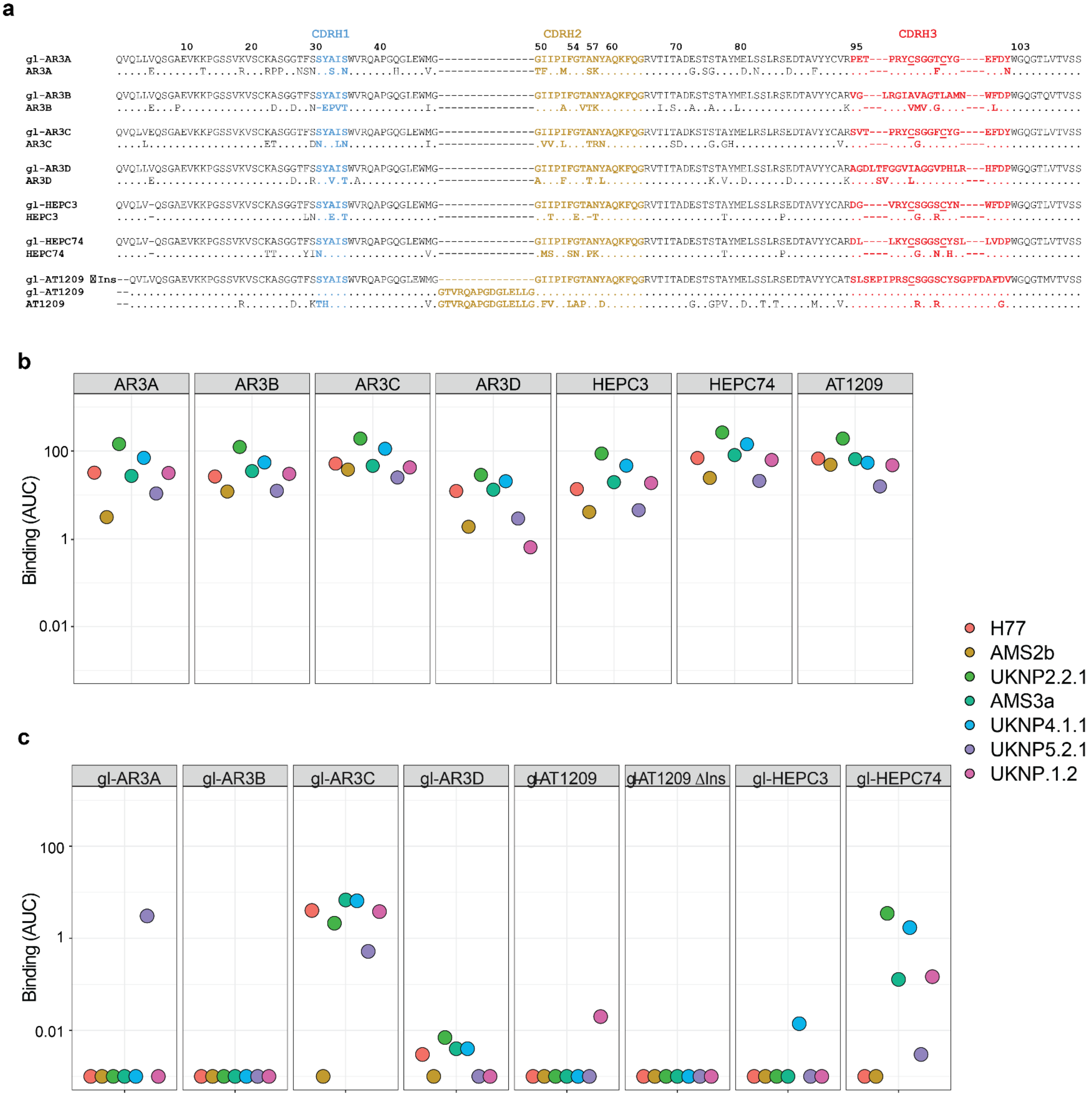
a
CDRH1
CDRH2
CDRH3
gl-AR3A
AR3A
gl-AR3B
AR3B
gl-AR3C
AR3C
gl-AR3D
AR3D
gl-HEPC3
HEPC3
gl-HEPC74
HEPC74
gl-AT1209 Ins
gl-AT1209
AT1209
b
AR3A
AR3B
AR3C
AR3D
HEPC3
HEPC74
AT1209
Binding (AUC)
100
1
0.01
H77
AMS2b
UKNP2.2.1
AMS3a
UKNP4.1.1
UKNP5.2.1
UKNP.1.2
c
gl-AR3A
gl-AR3B
gl-AR3C
gl-AR3D
gl-AT1209
gl-AT1209 ΔIns
gl-HEPC3
gl-HEPC74
Binding (AUC)
100
1
0.01

Supplementary Figure 1. Binding levels of AR3C-class bNAbs (a) Sequences of the HC of the different antibodies aligned. CDRs are color-labelled. Sequences are numbered using Kabat numbering. Cysteines involved in disulfide bond in the CDRH3 (CxGGxC) underlined. **(b)** Binding of mature AR3C-class bNAbs and **(c)** their inferred germline predecessors against seven different strains of HCV (genotypes 1-6). ELISA binding signals are represented as area under the curve (AUC) normalized to AP33 loading control (y-axis) for both germline and mature bNAbs (x-axis).

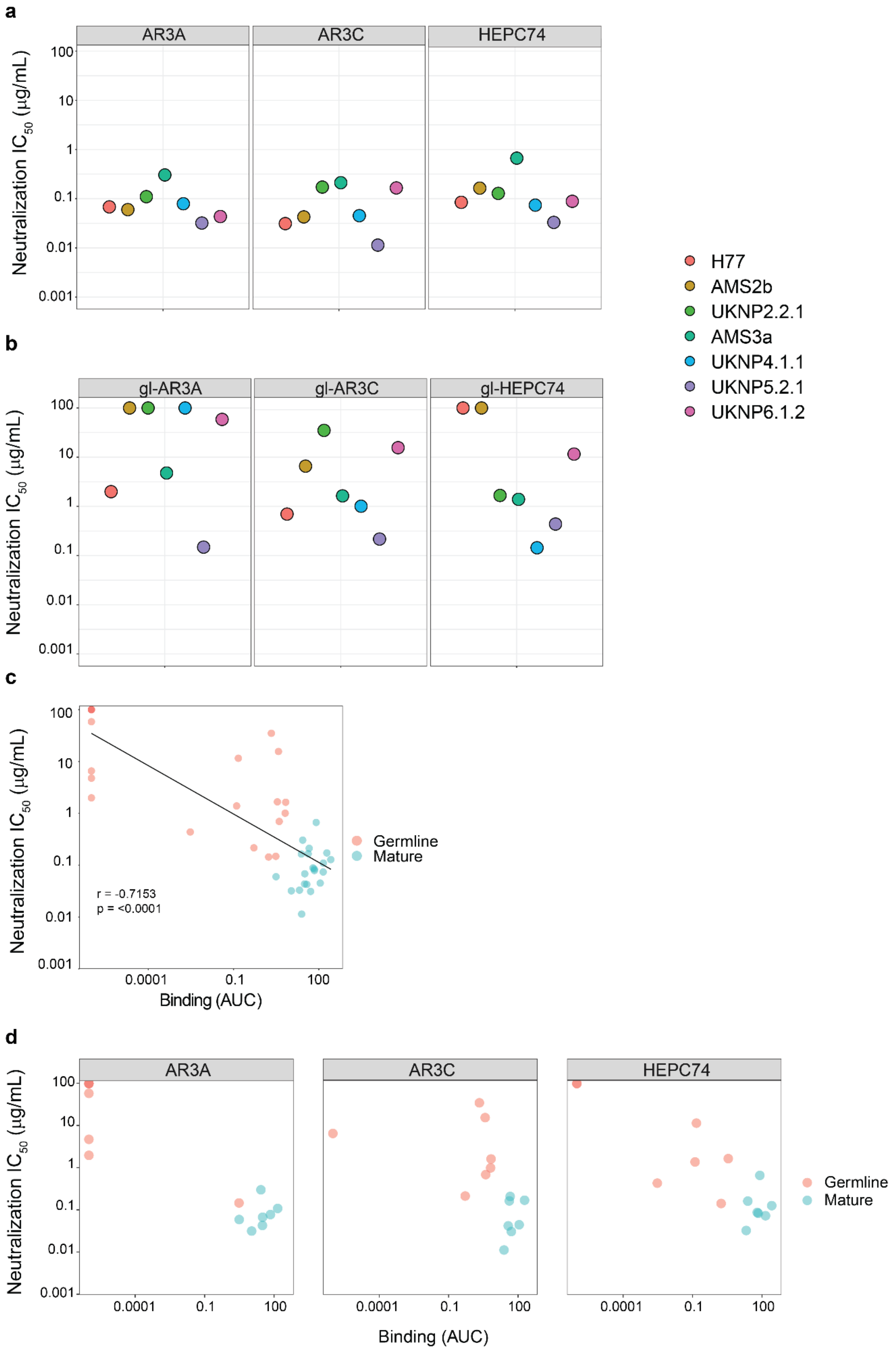
a
Neutralization IC50 (µg/mL)
AR3A
AR3C
HEPC74
100
10
1
0.1
0.01
0.001
H77
AMS2b
UKNP2.2.1
AMS3a
UKNP4.1.1
UKNP5.2.1
UKNP6.1.2
b
gl-AR3A
gl-AR3C
gl-HEPC74
c
r = -0.7153
p = <0.0001
0.0001
0.1
100
Binding (AUC)
Germline
Mature
d
Binding (AUC)

Supplementary Figure 2. Neutralization capacity of AR3C-class bNAbs. Neutralization IC_{50} titers of AR3A, AR3C and HEPC74 **(a)** and gl-AR3A, gl-AR3C and gl-HEPC74 **(b)** against HCVpp clones derived from seven different strains (genotypes 1-6). **(c)** A scatter plot of neutralization titer (IC_{50}) of AR3A, AR3C and HEPC74 and their germline precursors against HCVpp clones (y-axis) vs. binding (AUC) against the sequence-matched E2E1 trimers (x-axis). Spearman correlation test (r=-0.7153) and P (p=1.023e-07) values were computed in Rstudio (R version 4.0.4). **(d)** Individual cross-comparisons grouped per antibody from **(c)**.

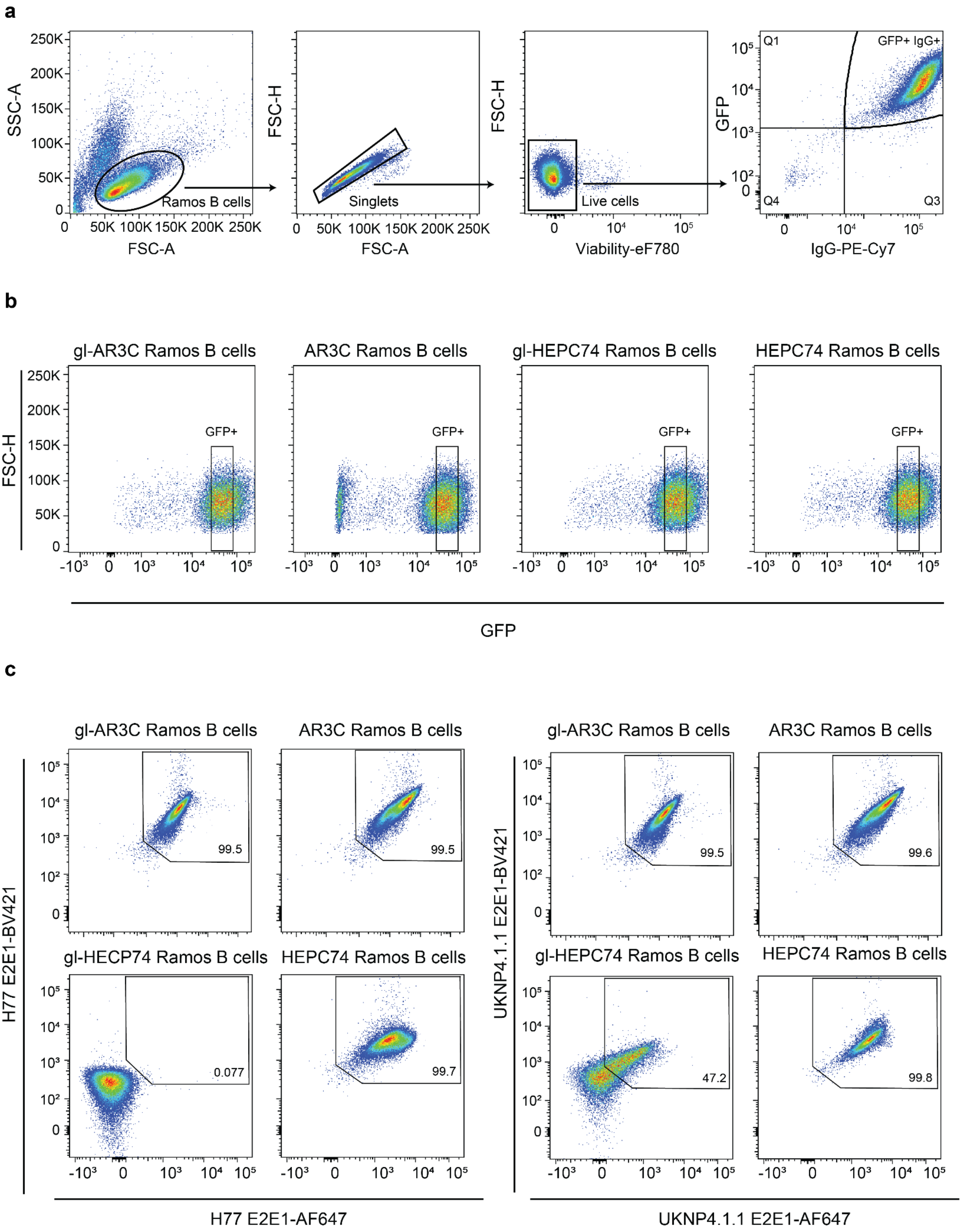
a
SSC-A
250K
200K
150K
100K
50K
0
FSC-A
Ramos B cells
FSC-H
Singlets
FSC-H
Live cells
Viability-eF780
GFP
Q1
GFP+ IgG+
Q4
Q3
IgG-PE-Cy7
b
gl-AR3C Ramos B cells
AR3C Ramos B cells
gl-HEPC74 Ramos B cells
HEPC74 Ramos B cells
FSC-H
GFP+
GFP
c
gl-AR3C Ramos B cells
AR3C Ramos B cells
gl-HECP74 Ramos B cells
HEPC74 Ramos B cells
H77 E2E1-BV421
99.5
99.5
0.077
99.7
H77 E2E1-AF647
gl-AR3C Ramos B cells
AR3C Ramos B cells
gl-HEPC74 Ramos B cells
HEPC74 Ramos B cells
UKNP4.1.1 E2E1-BV421
99.5
99.6
47.2
99.8
UKNP4.1.1 E2E1-AF647

Supplementary Figure 3. Selection and binding of generated (gl-)AR3C and (gl-)HEPC74 B cells to HCV E2E1 trimers. (a) Gating strategy to identify live (via-) B cells expressing the transduced BCR (GFP+/IgG+). Same as in Sliepen et al, Nat commun. **(b)** Selection scheme of IgG+ cells prior to calcium flux assay. Controlled levels of GFP expression for each cell line were selected to ensure similar levels of IgG across cell lines and proper understanding of the assay **(c)** Antigen specificity of Ramos B cells designed to express AR3C, gl-AR3C, HEPC74, or gl-HEPC74 to H77 (left) and UKNP4.1.1 (right) E2E1. The numbers inside the boxes represent the frequency (%) of cells in a gate.

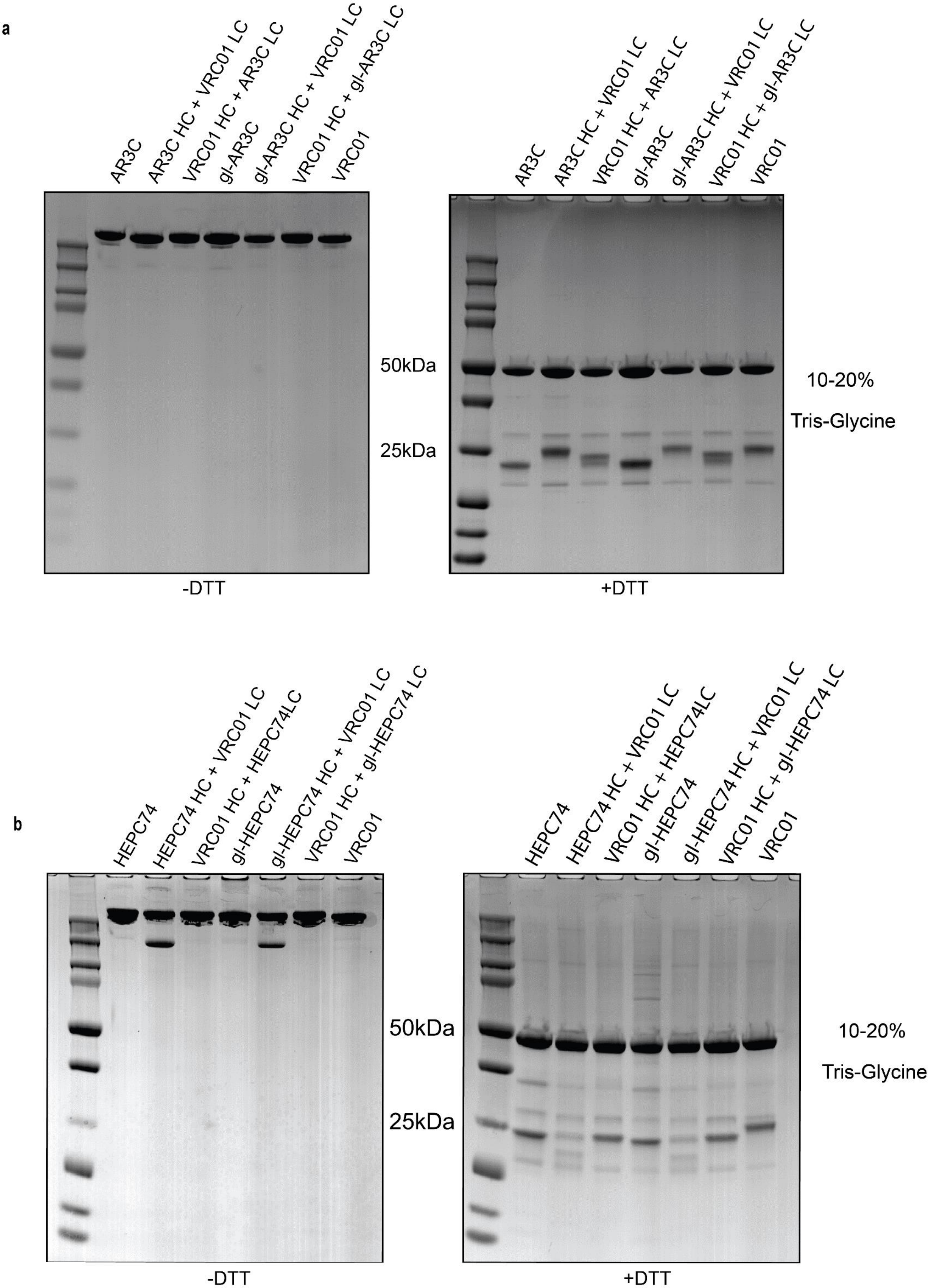

a
AR3C
AR3C HC + VRC01 LC
VRC01 HC + AR3C LC
gl-AR3C
gl-AR3C HC + VRC01 LC
VRC01 HC + gl-AR3C LC
VRC01
50kDa
25kDa
-DTT
+DTT
10-20%
Tris-Glycine
b
HEPC74
HEPC74 HC + VRC01 LC
VRC01 HC + HEPC74LC
gl-HEPC74
gl-HEPC74 HC + VRC01 LC
VRC01 HC + gl-HEPC74 LC
VRC01
50kDa
25kDa
-DTT
+DTT
10-20%
Tris-Glycine

Supplementary Figure 4. Chimeric antibody gels. Non-reducing SDS-PAGE gel (left) and reducing SDS-PAGE gel (right) analysis of **(a)** AR3C and **(b)** HECP74 antibodies and their chimeric versions combinations with VRC01 HC or LC. PageBlue Protein Staining Solution was used to visualize the purified antibodies. The 50 kDa and 25 kDa marks indicate the approximate apparent molecular weight of HC and LC, respectively.

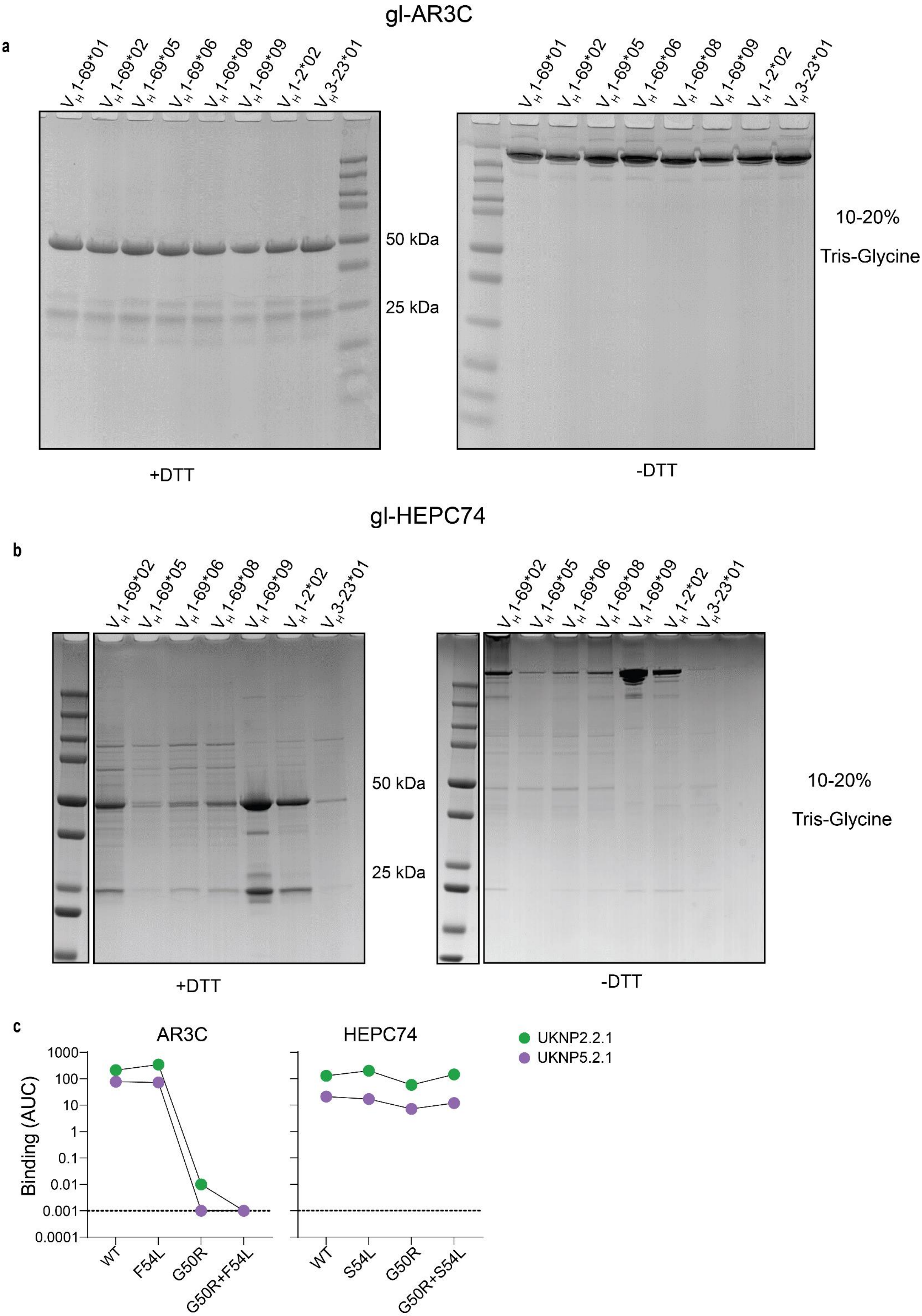
gl-AR3C
a
VH1-69*01
VH1-69*02
VH1-69*05
VH1-69*06
VH1-69*08
VH1-69*09
VH1-2*02
VH3-23*01
50 kDa
25 kDa
+DTT
VH1-69*01
VH1-69*02
VH1-69*05
VH1-69*06
VH1-69*08
VH1-69*09
VH1-2*02
VH3-23*01
10-20%
Tris-Glycine
-DTT
gl-HEPC74
b
VH1-69*02
VH1-69*05
VH1-69*06
VH1-69*08
VH1-69*09
VH1-2*02
VH3-23*01
50 kDa
25 kDa
+DTT
VH1-69*02
VH1-69*05
VH1-69*06
VH1-69*08
VH1-69*09
VH1-2*02
VH3-23*01
10-20%
Tris-Glycine
-DTT
c
AR3C
HEPC74
UKNP2.2.1
UKNP5.2.1
Binding (AUC)
1000
100
10
1
0.1
0.01
0.001
0.0001
WT
F54L
G50R
G50R+F54L
WT
S54L
G50R
G50R+S54L

Supplementary Figure 5. Reducing SDS-PAGE gel (left) and non-reducing SDS-PAGE gel analysis (right) of antibodies consisting of **(a)** gl-AR3C and **(b)** gl-HECP74 CDRH3 engrafted in different VH backgrounds. PageBlue Protein Staining Solution was used to visualize the purified antibodies. The 50 kDa and 25 kDa marks indicate the approximate apparent molecular weight of HC and LC, respectively. **(c)** ELISA binding of AR3C and HEPC74 or the same mAbs with CDRH2 mutations S/F54L, G50R or S/F54L+G50R to UKNP2.2.1 and UKNP5.2.1 E2E1 trimers . Binding is represented as AUC and the dotted line represents the detection limit.

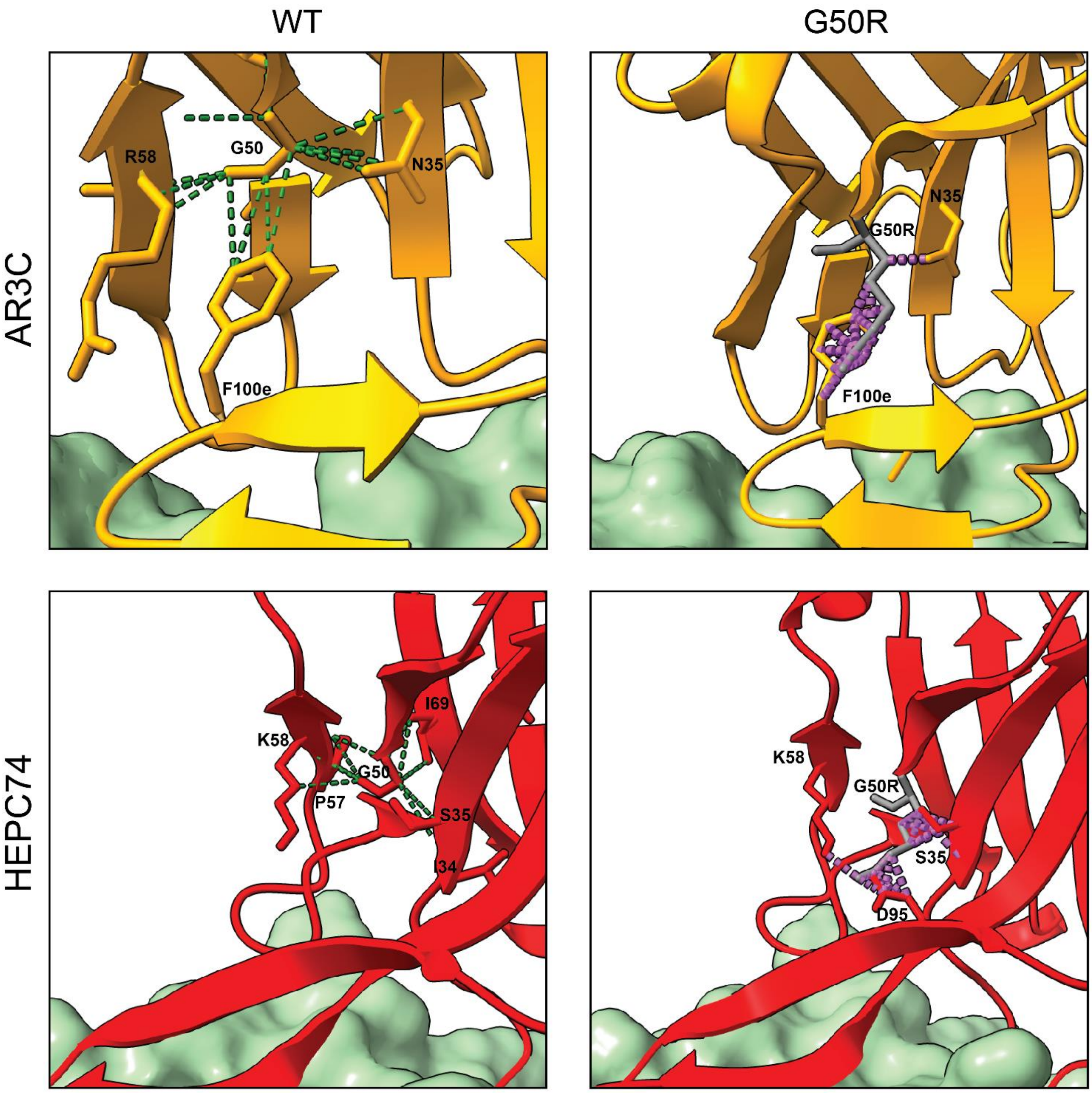

WT
G50R
AR3C
HEPC74
R58
G50
N35
F100e
G50R
N35
F100e
K58
I69
G50
P57
S35
I34
K58
G50R
S35
D95

Supplementary Figure 6. Modelled effect of G50R mutation on AR3C and HEPC74. Representation of interactions and clashes of G50R in AR3C (top, PDB: 4MWF) and HEPC74 (bottom, PDB:6MEH) Potential interactions between residues are shown in dotted green lines and in grey the mutation G50R is modelled with possible clashes in purple. Residues are labelled according to Kabat numbering.

a

Peptide	Sequence (AA)	Length	Mw (Da)
AR3C_CDRH3	RSVTPRY**C**GGGF**C**YGEFDY	19	2175
HEPC3_CDRH3	RDGVRY**C**GGGR**C**YNWFDP	18	2119
HEPC74_CDRH3	RDLLKY**C**GGGN**C**HSLLVD	18	1961

b

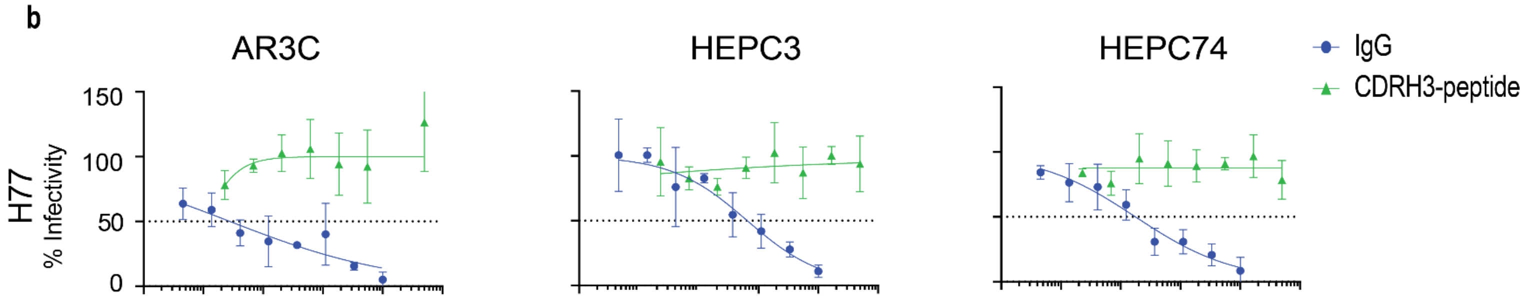

	IgG	CDRH3-peptide
IC50	0.03	NA

	IgG	CDRH3-peptide
IC50	0.65	NA

	IgG	CDRH3-peptide
IC50	0.18	NA

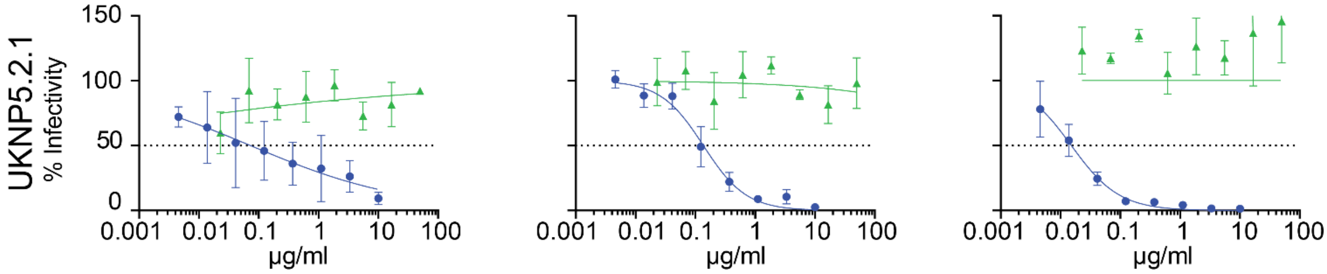

	IgG	CDRH3-peptide
IC50	0.07	NA

	IgG	CDRH3-peptide
IC50	0.14	NA

	IgG	CDRH3-peptide
IC50	0.02	NA

Supplementary Figure 7. CDRH3 soluble peptides. (a) Amino acid sequence, length and predicted molecular weight of of the AR3C, HEPC3 and HEPC74 CDRH3 peptides. In bold and underlined, the residues (cysteines) involved in disulfide bridge. **(b)** HCVpp neutralization by AR3C-class CRH3-based peptides. The prototypical lab strain H77 (top) and the clinical isolate UKNP5.2.1 (bottom) were incubated with a serial dilution of IgG or CDRH3 prior to infection. Data are expressed as percent infectivity relative to the wells not preincubated with antibody or peptide and each point is the mean of three replicate values. Error bars indicate the standard deviations between replicate wells. Data were fitted using the log(inhibitor) vs. response (four parameters) function on GraphPad Prism. The dotted line shows the 50% of infectivity (IC_{50}) value which are compiled under each graph. NA, not applicable (no curve could be fitted).

Supplementary Tables

Antibody	V_H gene	D gene	J_H gene	CDRH3 length
**AR3A**	1-69*01	2-15*01	4*02	20
**AR3B**	1-69*01	3-10*01	5*02	21
**AR3C**	1-69*06	2-21*01	4*02	20
**AR3D**	1-69*01	3-16*02	5*02	24
**HEPC3**	1-69*01	2-15*01	5*02	19
**HEPC74**	1-69*01	2-15*01	5*02	20
**AT1209**	1-69*01	2-15*01	3*01	27

Supplementary Table 1. Genetic characteristics of AR3C-class bNAbs. VH, D and JH gene usage and CDRH3 length for AR3A, AR3B, AR3C, AR3D, HEPC3, HEPC74, AT1209.

Supplementary Data – Uncropped gels

Supplementary Figure 4a

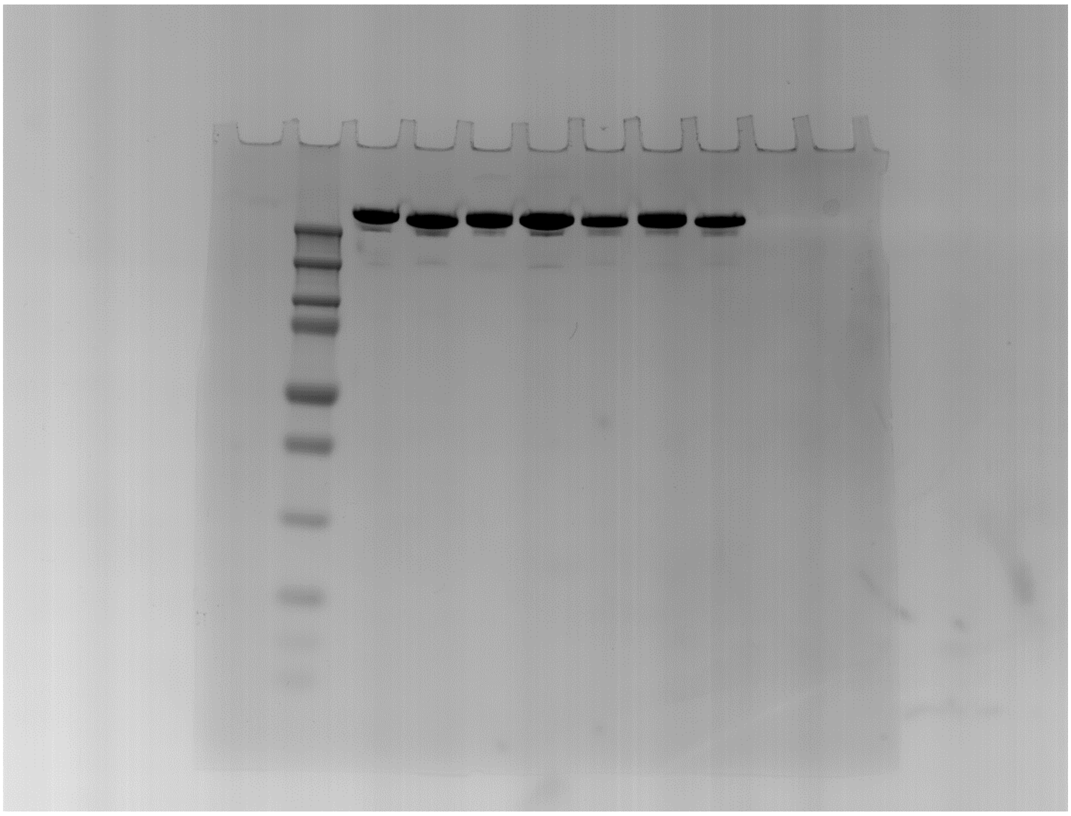

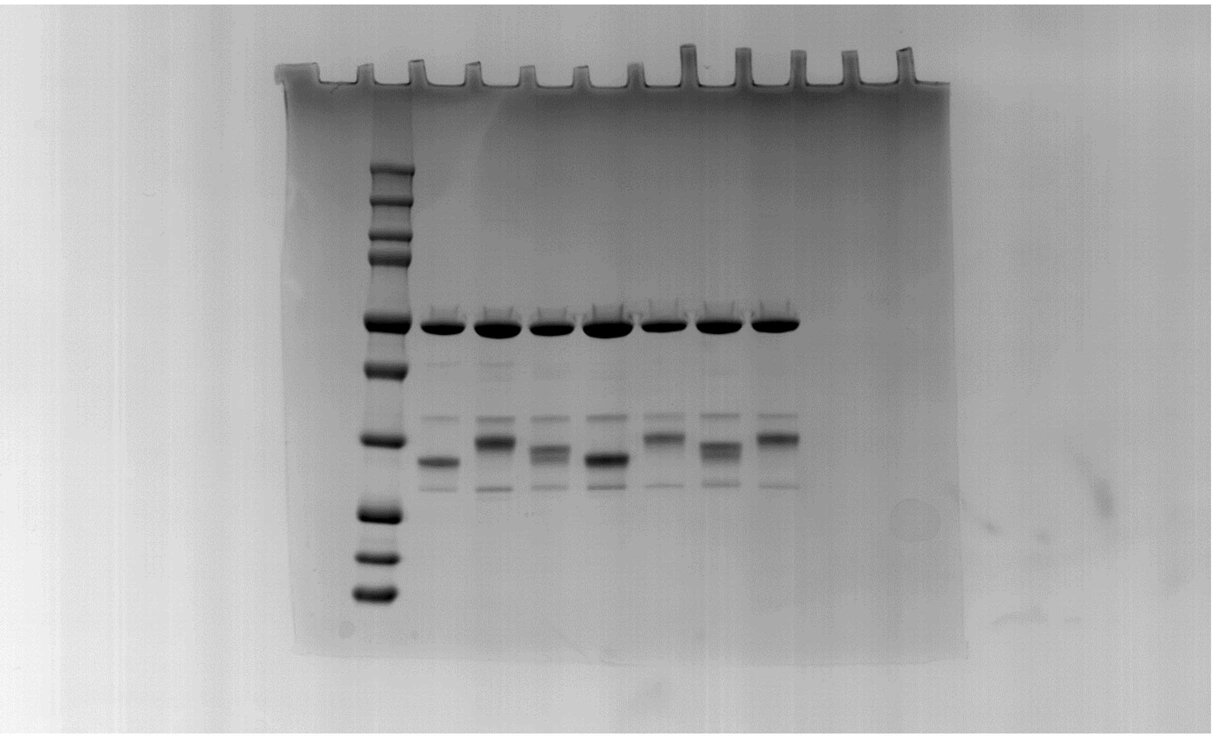

Supplementary Figure 4b

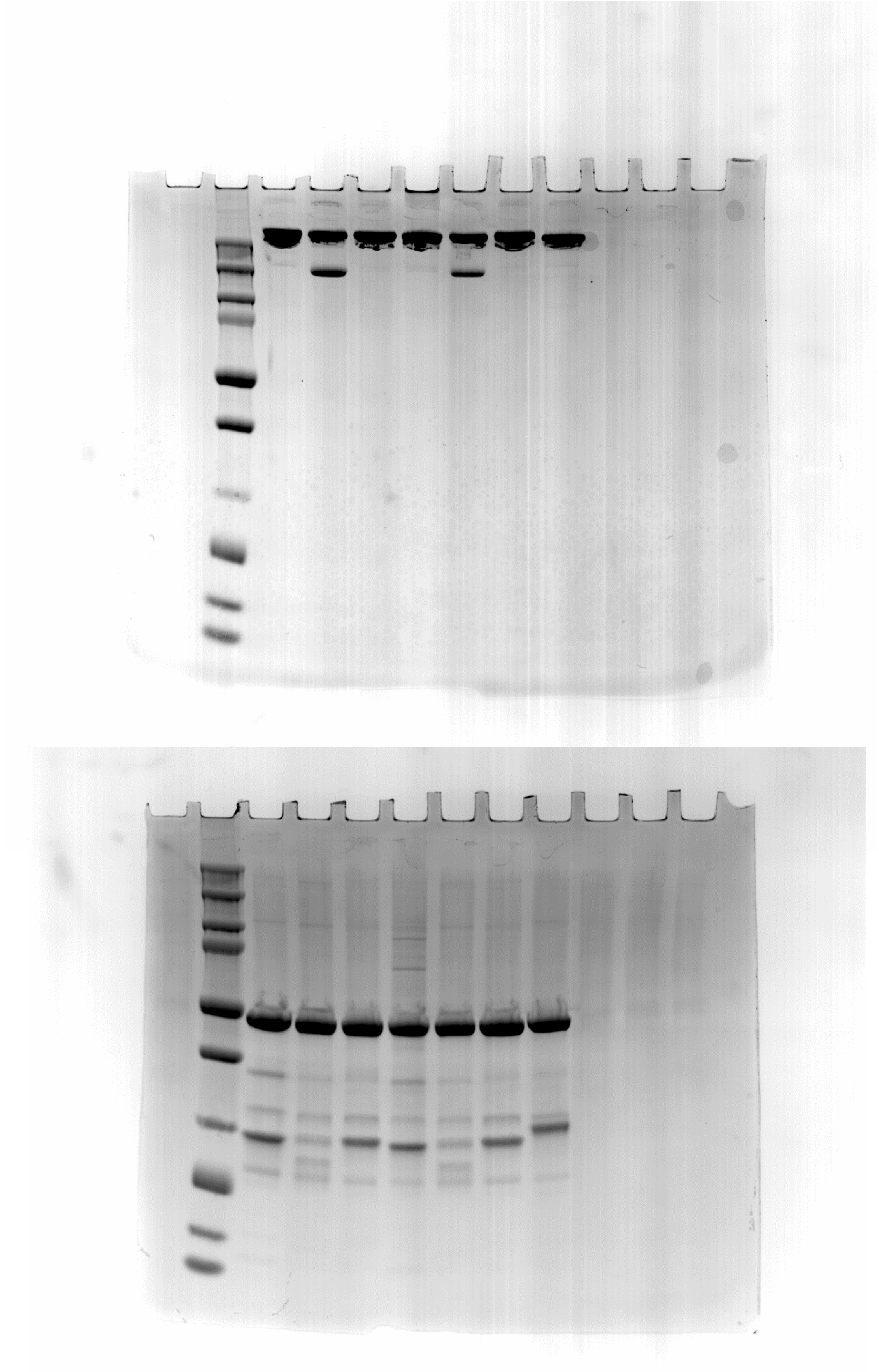

Supplementary Figure 5a

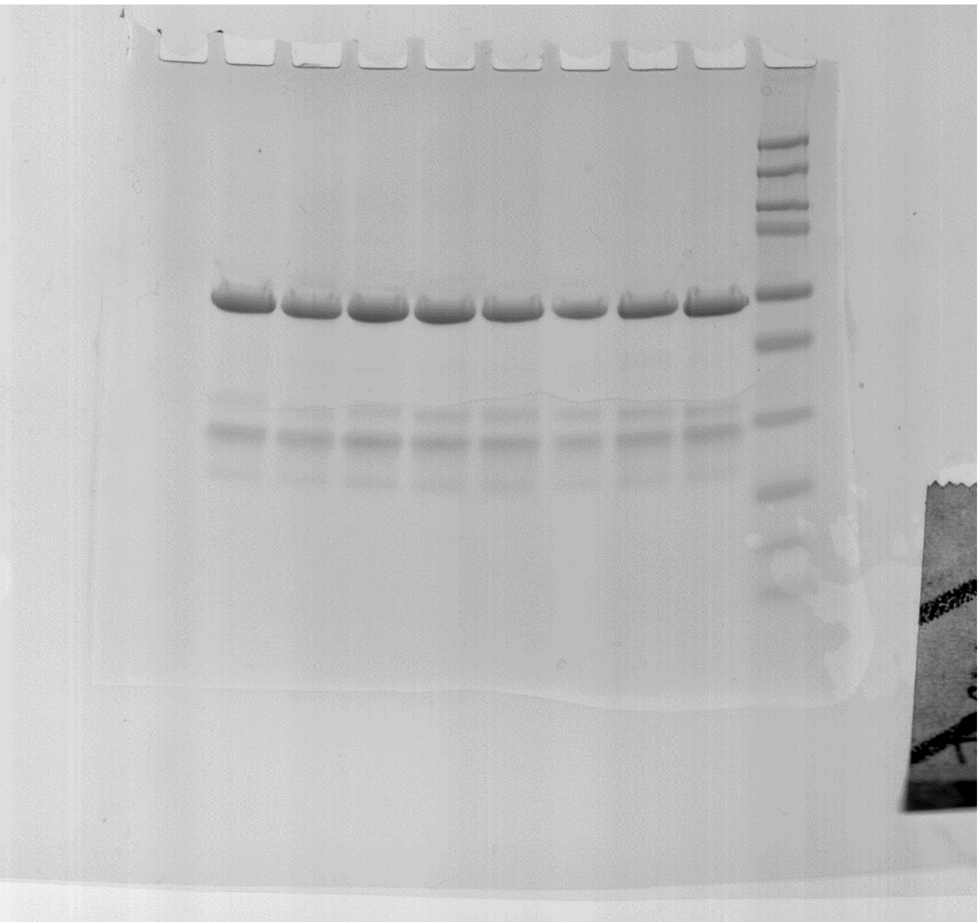

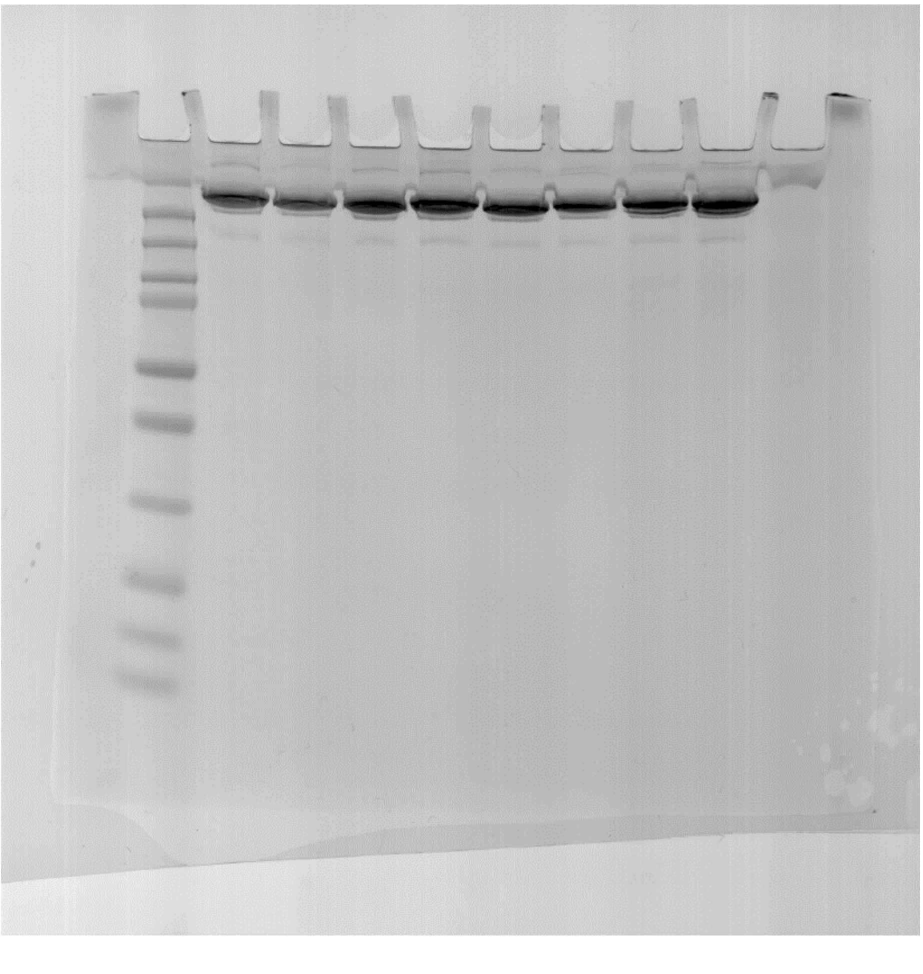

Supplementary Figure 5b

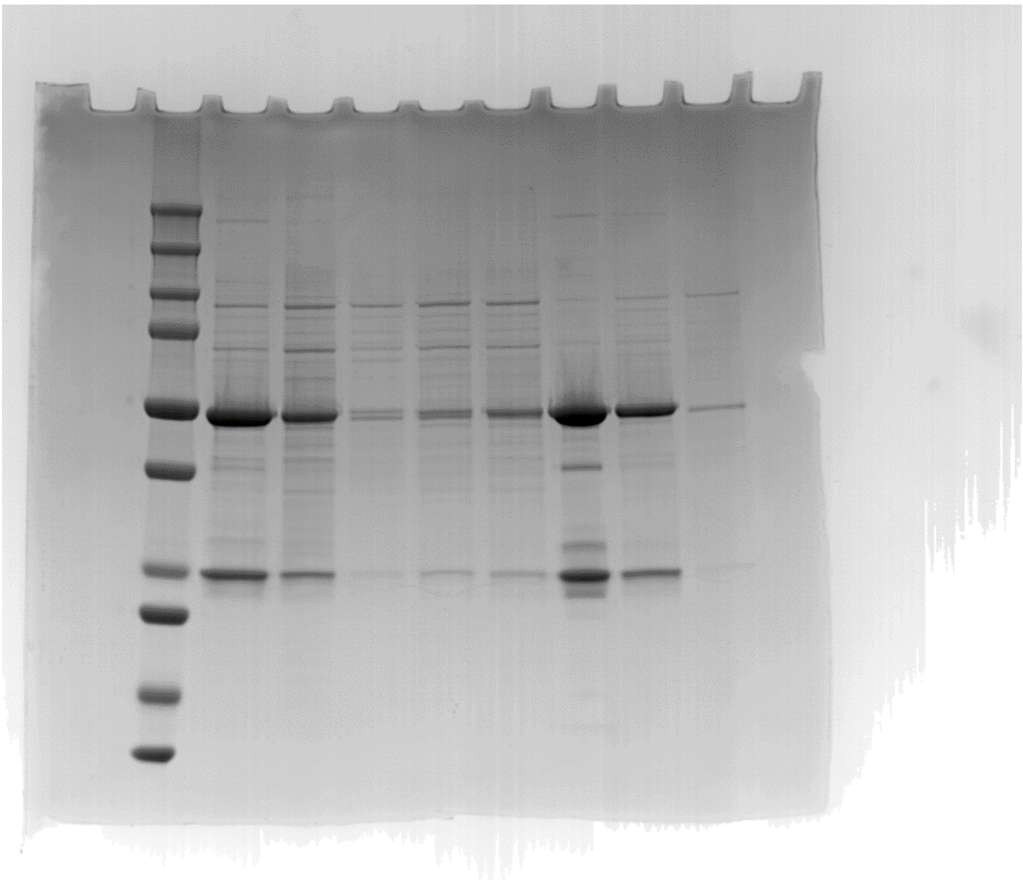

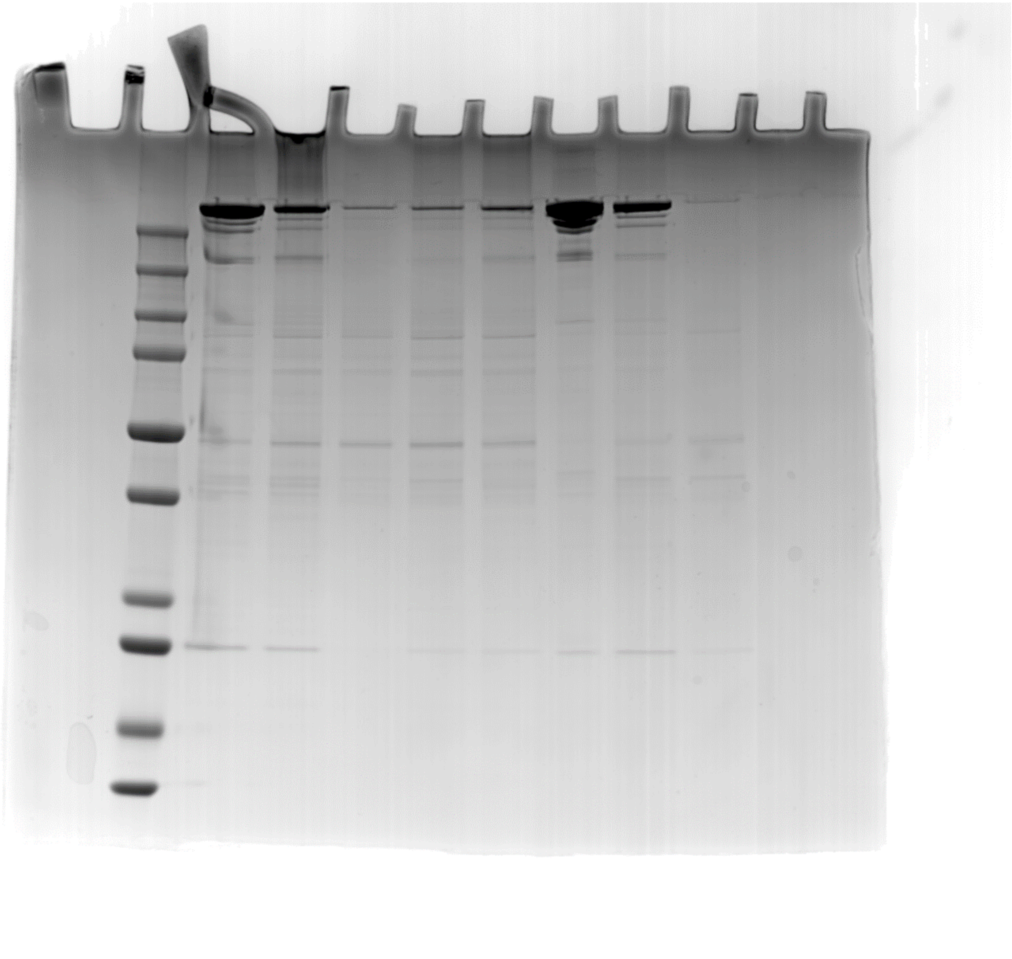

Supplement: Supplementary file 1 — Supplementary information [file 41467_2023_39690_MOESM1_ESM.pdf]
