# Supplementary material for: Signatures of VH1-69-derived hepatitis C virus neutralizing antibody precursors defined by binding to envelope glycoproteins
Source: Nat Commun. 2023 Jul 7;14:4036. doi: 10.1038/s41467-023-39690-0 (PMC10328973; doi:10.1038/s41467-023-39690-0)
Supplement: Supplementary file 3 — Reporting Summary [file 41467_2023_39690_MOESM3_ESM.pdf]

nature portfolio

Corresponding author(s): Kwinten Sliepen
Rogier W. Sanders

Last updated by author(s): Nov 15, 2022

Reporting Summary

Nature Portfolio wishes to improve the reproducibility of the work that we publish. This form provides structure for consistency and transparency in reporting. For further information on Nature Portfolio policies, see our Editorial Policies and the Editorial Policy Checklist.

Statistics

For all statistical analyses, confirm that the following items are present in the figure legend, table legend, main text, or Methods section.

n/a	Confirmed	
☒	☐	The exact sample size (*n*) for each experimental group/condition, given as a discrete number and unit of measurement
☒	☐	A statement on whether measurements were taken from distinct samples or whether the same sample was measured repeatedly
☐	☒	The statistical test(s) used AND whether they are one- or two-sided<br>*Only common tests should be described solely by name; describe more complex techniques in the Methods section.*
☐	☒	A description of all covariates tested
☐	☒	A description of any assumptions or corrections, such as tests of normality and adjustment for multiple comparisons
☐	☒	A full description of the statistical parameters including central tendency (e.g. means) or other basic estimates (e.g. regression coefficient) AND variation (e.g. standard deviation) or associated estimates of uncertainty (e.g. confidence intervals)
☐	☐	For null hypothesis testing, the test statistic (e.g. *F*, *t*, *r*) with confidence intervals, effect sizes, degrees of freedom and *P* value noted<br>*Give P values as exact values whenever suitable.*
☒	☐	For Bayesian analysis, information on the choice of priors and Markov chain Monte Carlo settings
☒	☐	For hierarchical and complex designs, identification of the appropriate level for tests and full reporting of outcomes
☐	☒	Estimates of effect sizes (e.g. Cohen's *d*, Pearson's *r*), indicating how they were calculated

Our web collection on statistics for biologists contains articles on many of the points above.

Software and code

Policy information about availability of computer code

Data collection	We used Microsoft Excel 2016 (16.05356.1000) for data storage.
Data analysis	Graphpad Prism 8.3, RStudio 1.2.5033 (R4.0.3), IMGT/V-QUEST software tool, Microsoft Excel 2016 (16.05356.1000), FlowJo v10.8.1, Power Point 2016 (16.05356.1000), Adobe Illustrator (27.6.1), AlphaFold-2.2.0 Multimer, UCSF ChimeraX (version 1.4)

For manuscripts utilizing custom algorithms or software that are central to the research but not yet described in published literature, software must be made available to editors and reviewers. We strongly encourage code deposition in a community repository (e.g. GitHub). See the Nature Portfolio guidelines for submitting code & software for further information.

Data

Policy information about availability of data

All manuscripts must include a data availability statement. This statement should provide the following information, where applicable:

- Accession codes, unique identifiers, or web links for publicly available datasets
- A description of any restrictions on data availability
- For clinical datasets or third party data, please ensure that the statement adheres to our policy

All data on the human BCR repertoire can be found at https://github.com/briney/grp_paper. Designed constructs are based on sequences deposited at GenBank: H77 (AAB67037; mutations R564C, V566A, G650E), UKNP2.1.1 (KU285220.1), AMS2b (KR094963.1), AMS3a (KR094964.1), UKNP4.1.1 (ALV85530.1), UKNP5.2.1 (ALV85536.1), and UKNP6.1.2 (ALV85538.1). Structures of AR3C-class bNAbs AR3C, AR3A, AR3B, AR3D, HEPC3, HEPC74 and AT1209 are publicly available (PDB

entries: 4MWF, 6BKB, 6BKC 6BKD 6MEI, 6MEH, 7T6X, respectively) as well as the E1E2 complex (PDB: 7T6X). Source Data are provided with this paper. Other data that support the findings of this study are available from the corresponding authors (K.S. and R.W.S.) upon reasonable request.

Human research participants

Policy information about studies involving human research participants and Sex and Gender in Research.

Reporting on sex and gender	N/A see below
Population characteristics	N/A see below
Recruitment	n/A see below
Ethics oversight	N/A

Note that full information on the approval of the study protocol must also be provided in the manuscript.

Field-specific reporting

Please select the one below that is the best fit for your research. If you are not sure, read the appropriate sections before making your selection.

☒ Life sciences ☐ Behavioural & social sciences ☐ Ecological, evolutionary & environmental sciences

For a reference copy of the document with all sections, see nature.com/documents/nr-reporting-summary-flat.pdf

Life sciences study design

All studies must disclose on these points even when the disclosure is negative.

Sample size	Binding assays were performed with the panel of antibodies and germline predecessors described in the manuscript against the panel of proteins available (also described). The use of smaller samples size was determined by the lack of binding of the antibodies to the proteins. Likewise, neutralization experiments were performed based on binding capacity. The analysis of the dataset was based on the available data from the dataset that we were capable of analyzing (i.e. 8 donors). Two donors were left out due to the large size of their individual dataset and the limitations of data processing in our lab. Specific sample size calculations were not performed.
Data exclusions	Some values on figure 1c were excluded. The exclusion was performed with outliers that made the calculations of the experiment not reliable.
Replication	Experiments were performed in duplo or triplo and/or repeated at least twice. All attempts to repeat the experiments were successful.
Randomization	No randomization could be applied the output of the experiments could not be biased.
Blinding	Blinding was not relevant for our studies.

Reporting for specific materials, systems and methods

We require information from authors about some types of materials, experimental systems and methods used in many studies. Here, indicate whether each material, system or method listed is relevant to your study. If you are not sure if a list item applies to your research, read the appropriate section before selecting a response.

Materials & experimental systems

n/a	Involved in the study	
☐	☒	Antibodies
☐	☒	Eukaryotic cell lines
☒	☐	Palaeontology and archaeology
☒	☐	Animals and other organisms
☒	☐	Clinical data
☒	☐	Dual use research of concern

Methods

n/a	Involved in the study	
☒	☐	ChIP-seq
☐	☒	Flow cytometry
☒	☐	MRI-based neuroimaging

Antibodies

Antibodies used	All antibodies were produced in the lab sing transient transfection using plasmids expressing the antibody heavy and light chains.

Antibodies used	Goat-anti-human IgG Jackson Immunoresearch (cat# 109-035-170) and used at a dilution 1:3000.<br>PE-Cy™7 Mouse Anti-Human IgG (#561298) - BD Biosciences - clone G18-145<br>Viability marker (eBioscience™ Fixable Viability Dye eFluor™ 780)
Validation	AR3C-class antibodies were validated by sequencing.<br>Goat-anti-human IgG was autheticated by manufacturer (https://www.jacksonimmuno.com/catalog/products/109-035-170)<br>IgG PE-Cy7 was validated by the manufacturer (https://www.bdbiosciences.com/en-nl/products/reagents/flow-cytometry-reagents/research-reagents/single-color-antibodies-ruo/pe-cy-7-mouse-anti-human-igg.561298). Antibody was titrated for best ratio saturation/noise.<br>Viability marker (eBioscience™ Fixable Viability Dye eFluor™ 780) was validated by manufacturer and titrated for best ratio saturation/noise (https://www.thermofisher.com/order/catalog/product/65-0865-14).

Eukaryotic cell lines

Policy information about cell lines and Sex and Gender in Research

Cell line source(s)	HEK293T and HEK293F cell lines were obtained from ATCC or Invitrogen, respectively.<br>Ramos B cells were obtained from dr. Li Wu and dr. Vineet N. KewalRamani via the NIH AIDS Reagent Program.<br>Huh-7 were a kind gift from François-Loïc Cosse.
Authentication	Ramos cell lines, HEK293T, and HEK293F were authenticated by the provider (ThermoFisher) and Ramos cells phenotype was validated by FACS, by binding to E2E1 probes (Supplementary Figure 3C). Huh-7 were authenticated by provider.
Mycoplasma contamination	Mycoplasma contamination All cell lines were tested negative for mycoplasma
Commonly misidentified lines<br>(See ICLAC register)	No commonly misidentiefied cell lines were used

Flow Cytometry

Plots

Confirm that:

☒ The axis labels state the marker and fluorochrome used (e.g. CD4-FITC).

☒ The axis scales are clearly visible. Include numbers along axes only for bottom left plot of group (a 'group' is an analysis of identical markers).

☒ All plots are contour plots with outliers or pseudocolor plots.

☒ A numerical value for number of cells or percentage (with statistics) is provided.

Methodology

Sample preparation	IgM-negative Ramos B cell lines were previously transduced with AR3C, HEPC74, gl-AR3C and gl-HEPC74 as described in the methods. Cells were then sorted for GFP+ IgG + IgM- using a FACS Aria-II SORP (BD Biosciences). B cells were expanded and cultured indefinitely. Cells were frozen for future use. For antigen specificity and Calcium flux assays, labelled probes and nonlabelled proteins were used as described in Methods and analysed using LSRFortessaTM(BD Biosciences).
Instrument	Aria-II SORP (BD Biosciences) and LSRFortessaTM (BD Biosciences).
Software	FlowJo v10.8.1. Data visualization with Graphpad Prism 8.3
Cell population abundance	Single cell line was used (so 100%).
Gating strategy	All cell lines were sorted as indicated in hte gating strategy of supplementary figure 3

☒ Tick this box to confirm that a figure exemplifying the gating strategy is provided in the Supplementary Information.
